# Greater occipital nerve block in management of chronic migraine; exploring clinical effectiveness and patient experience

**DOI:** 10.1186/1129-2377-14-S1-P65

**Published:** 2013-02-21

**Authors:** JC Williamson, J Winston, R De Silva

**Affiliations:** 1 Essex Centre for Neurosciences, UK

## 

Greater Occipital Nerve (GON) Block is a recognized treatment modality in the management of chronic migraine. Our study aimed to assess the patient experience and clinical effect of this procedure in 20 patients undergoing this intervention. 20 patients with a diagnosis of chronic migraine were admitted from the outpatient department for GON block procedure. Patients were asked to complete a pre-procedure questionnaire. This questionnaire focused on four domains: 1. Duration of symptoms, 2. Pre procedure ‘Headache Impact Test’ (HIT-6) score, 3. An objective pain score of current pain at time of procedure, 4. Subjective description of current pain character at the time of procedure. 20 minutes following the procedure patients were asked to repeat the objective and subjective assessment of current pain. At a one-month interval patients completed a repeat HIT-6 score.

## Summary of key findings

Only 40% of patients reported a reduction in pain score 20 minutes post procedure. 2. A majority of patients (85%) reported a change in the subjective nature of the pain 20 minutes post procedure 3. A majority of patients (80%) scored a lower HIT-6 score one month following procedure then prior to procedure. Average score difference was 5. 4. There was no clear correlation between symptom duration and reported improvement in immediate and one month assessment of headache. We hope to present full details of patient demographics, breakdown of pre and post procedure HIT-6 scores as well as patient feedback of their experience of undergoing a GON block procedure.

